# Incident HIV acquisition among pregnant women in Botswana: findings from the Tsepamo birth outcomes surveillance study

**DOI:** 10.1002/jia2.26008

**Published:** 2023-01-24

**Authors:** Aamirah Mussa, Gloria Katuta Mayondi, Modiegi Diseko, Judith Mabuta, Mompati Mmalane, Joseph Makhema, Shahin Lockman, Chelsea Morroni, Roger Shapiro, Rebecca Zash

**Affiliations:** ^1^ Botswana‐Harvard AIDS Institute Partnership Gaborone Botswana; ^2^ Usher Institute University of Edinburgh Edinburgh UK; ^3^ Brigham and Women's Hospital Boston Massachusetts USA; ^4^ Harvard T.H. Chan School of Public Health Boston Massachusetts USA; ^5^ MRC Centre for Reproductive Health and Centre for Global Health University of Edinburgh Edinburgh UK; ^6^ Beth Israel Deaconess Medical Center Boston Massachusetts USA

**Keywords:** HIV, seroconversion, vertical transmission, pregnancy, Botswana, Africa

## Abstract

**Introduction:**

In Botswana, where almost all pregnant women known to have HIV receive antiretroviral therapy, a large proportion of vertical HIV transmission may occur among women with incident undiagnosed HIV infection during pregnancy. Botswana guidelines recommend repeat HIV testing every 3 months in pregnancy, with at least one test in the third trimester. We evaluated the rate of repeat HIV testing, calculated HIV incidence during pregnancy and estimated missed seroconversions.

**Methods:**

In the Botswana Tsepamo Study, we abstracted HIV test dates and results from obstetric records of all women who delivered at maternity wards in 18 communities between 7th May 2017 and 20th August 2021. We defined seroconversion as an initial negative/indeterminate HIV test in pregnancy followed by a positive test during pregnancy/at delivery. The incidence rate (IR) of seroconversion was calculated among women with > = 2 known test dates. Missed seroconversions were estimated among women without a test in the third trimester by applying the IR to the time after the last HIV test until delivery.

**Results:**

Among 103,529 women delivering in the study period testing negative at the first test and with known conception and HIV test dates, 29,085 (28%) were tested in one trimester of pregnancy, 73,156 (71%) were tested in ≥ 2 trimesters of pregnancy and 9628 (9%) had a test in all trimesters. A total of 78,162 (75%) women had a third‐trimester test. There were 223 seroconversions (2.58/1000 pregnancies, 0.26%) among those with ≥ 2 known HIV test dates, yielding an IR of 0.69/100 person‐years. Among 25,289 women who did not have a test in the third trimester, we estimate approximately 58 seroconversions may have been missed during pregnancy due to a lack of repeat testing. Factors associated with seroconversion during pregnancy included younger age, less education and not being married.

**Conclusions:**

More than two‐thirds of women had repeat HIV testing in pregnancy and HIV incidence was low. However, an estimated 21% of seroconversions in pregnancy were likely missed due to a lack of re‐testing. To reach the goal of zero new paediatric HIV infections, Botswana will need to intensify repeat HIV testing in the third trimester of pregnancy.

## 1 INTRODUCTION

Vertical transmission of HIV continues to be a major global health challenge, as the majority of paediatric HIV infections are due to transmission during pregnancy, delivery and breastfeeding [1]. In the absence of interventions, it is estimated that the rate of vertical transmission is between 15% and 40% [2], but the risk is even higher in the setting of acute HIV seroconversion in pregnancy [3]. As access to antiretroviral therapy (ART) for women with diagnosed HIV increases globally, HIV seroconversion during pregnancy is increasingly becoming a significant contributor towards vertical transmission [4, 5, 6]. Routine HIV testing during pregnancy, with repeat testing in the third trimester, is, therefore, critical for timely identification of seroconversion, initiation of ART during the intra‐partum period and identification of infants who are at high risk of vertical HIV transmission, to allow for close monitoring and infant prophylaxis. Current WHO guidelines recommend repeat testing of women with an HIV negative or unknown status during pregnancy in high HIV burden settings to prevent vertical transmission [7].

In 1999, the Botswana government launched its prevention of mother‐to‐child transmission programme, the first of its kind in Africa [8]. Since then, a multifaceted approach of testing, treatment and prevention has succeeded in reducing HIV incidence in Botswana, with annualized incidence per 1000 population (all ages) declining from 12.6 in 1990 to 4.4 in 2020 [9]. Almost all pregnant women with known HIV in Botswana receive ART, and uptake of HIV testing during pregnancy is high (>95%), with testing routinely performed at antenatal care (ANC) visits during pregnancy [10, 11, 12]. Botswana was recently recognized as the first high HIV burden country to reach a key milestone in the elimination of vertical transmission of HIV [13]. This certification is granted by the WHO to countries which have achieved vertical transmission rates of <5%, provision of ANC and antiretroviral treatment to >90% of pregnant women, and an HIV case rate of <500 per 100,000 live births [13]. In line with WHO guidelines, current Botswana guidelines recommend repeat HIV testing every 3 months in pregnancy, with documented results in the third trimester or at delivery [11]. However, no studies have evaluated compliance with these guidelines. Identifying the risk of incident HIV acquisition among pregnant women in Botswana and exploring rates and timing of antenatal HIV testing are essential for informing prevention interventions, which are critical to decrease vertical transmission.

Using data from a large ongoing birth outcomes surveillance study in Botswana (the Tsepamo Study), we determined the number and timing of HIV tests among pregnant women in Botswana, calculated HIV incidence and estimated the number of missed seroconversions. We also identified factors associated with HIV seroconversion during pregnancy.

## 2 METHODS

We performed an analysis of data collected in the ongoing Tsepamo birth outcomes surveillance study, which started in August 2014, and collects data from antenatal and obstetric records of women who delivered live‐born and stillborn infants in public maternity wards in Botswana. We used data collected from women who gave birth at 18 public maternity wards (2 cities, 2 towns and 14 villages) between 7th May 2017 and 20th August 2021, accounting for approximately 70% of all births in Botswana during this period. More than 95% of pregnant women in Botswana deliver in a health facility. In the Tsepamo study, data are collected at the time of discharge from the postnatal ward and entered into the study database. Information, including demographic data, maternal medical history, maternal diagnoses and laboratory results during pregnancy, is extracted from the obstetric record. Detailed study methods have been previously described [12]. Using this database, we abstracted HIV test dates, HIV test results and demographic data. HIV diagnosis in Botswana is made using a point‐of‐care enzyme immunoassay screening test (the third‐generation Determine^TM^ HIV‐1/2). If the test is negative, the patient is considered HIV negative. If positive, a confirmatory test (the third‐generation Uni‐Gold^TM^ HIV‐1/2) is performed.

Seroconversion was defined as an initial negative or indeterminate HIV test during pregnancy followed by a positive test during pregnancy or at delivery. A minimum of 72 hours between an indeterminate test and a positive test was required to be classed as a seroconversion. The incidence rate (IR) of seroconversion was estimated among women with > = 2 known HIV test dates during pregnancy, with a first negative/indeterminate test. The IR was calculated by dividing the number of seroconversions during pregnancy by the total person‐time between the last negative/indeterminate HIV test and the mid‐point between the last negative/indeterminate test and the first positive HIV test (for women who seroconverted) or total person‐time between initial negative/indeterminate test and last test during pregnancy or within 7 days of delivery (for non‐seroconverters). Missed seroconversions were estimated among women without a test in the third trimester by applying the IR to the time after their last HIV test until delivery or duration of their whole pregnancy for women who did not have a documented test during pregnancy. A secondary analysis was performed that included tests performed at or within 7 days after delivery as non‐missing, as while this group represents a missed opportunity to reduce *in‐utero* vertical transmission, there is an opportunity for intra‐partum/infant prophylaxis.

The estimated gestational age at delivery was documented by midwives at the time of delivery and was based upon the estimated date of delivery calculated during ANC (estimated using last menstrual period [LMP] and confirmed by ultrasonography, when available, and fundal height measurements when neither LMP nor ultrasonography was available). The estimated date of conception was calculated by subtracting the estimated gestational age (weeks) at delivery from the delivery date. The number of tests was extracted from the obstetric record and was reported among women with an initial negative/indeterminate HIV test during pregnancy. The timing of the HIV tests was defined as the number of days between the estimated date of conception and the date of the HIV test. HIV testing conducted between 4 weeks prior to the date of conception to the end of week 12 of pregnancy was counted as a first‐trimester test. A second‐trimester test was defined as a test between week 13 and the end of week 26, and a third‐trimester test was a test between week 27 up to but not including the date of delivery.

To identify factors associated with incident HIV during pregnancy, we performed logistic regression analysis, where the outcome variable was incident HIV during pregnancy. Co‐variates included age, education, marital status, occupation, parity, gravida, region and area. All analyses were performed using Stata (Version 16.1, StataCorp, College Station, TX).

Ethical approval, with a waiver of informed consent for de‐identified surveillance data, was obtained from the Botswana Health Research Development Committee and the Office for Human Research Administration at Harvard T.H. Chan School of Public Health.

## 3 RESULTS

**Figure 1:**
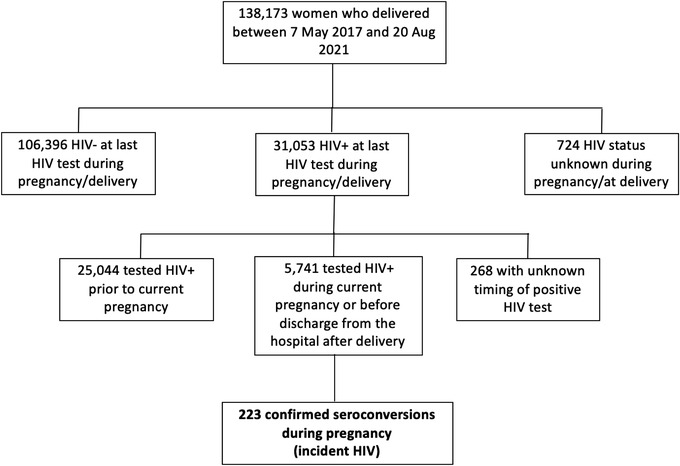
Flow chart of deliveries, HIV status and seroconversions identified in the Tsepamo study during the study period.

A total of 138,173 women delivered at participating sites between 7th May 2017 and 20th August 2021, of whom 106,396 (77%) were HIV negative at the last test during pregnancy/delivery, 31,053 (22%) were living with HIV and 724 (1%) had an unknown HIV status. Among those living with HIV at delivery, 25,044 (81%) tested positive prior to their current pregnancy, 268 (1%) had an unknown timing of positive HIV test and 5741 (18%) tested positive for the first time during their current pregnancy or before discharge from the hospital after delivery; within this group with newly identified HIV acquisition, 223 (4%) were new seroconversions in pregnancy (Figure 1).

Socio‐economic and demographic characteristics are shown in Table 1. The median age at delivery was 26 (IQR 22, 32). Most women reported being single (86%), unemployed/housewife (58%), having a secondary level of education (69%) and living in a rural area (65%).

**Table 1:** Socio‐economic and demographic characteristics of women in the Tsepamo study who delivered during the study period

	All women	HIV negative at last HIV test during pregnancy/delivery	Women who seroconverted during pregnancy
	*n* = 138,173	*n* = 106,396	*n* = 223
Age	26 (22–32)	25 (21–31)	23 (20–28)
Marital status			
Single	118,962 (86.10)	91,366 (85.87)	209 (93.72)
Married	13,901 (10.06)	11,230 (10.55)	7 (3.14)
Widowed/divorced	414 (0.30)	229 (0.22)	2 (0.90)
Unknown	4896 (3.54)	3571 (3.36)	5 (2.24)
Occupation			
Student	7471 (5.41)	6858 (6.45)	12 (5.38)
Unemployed/housewife	79,982 (57.89)	61,006 (57.34)	145 (65.02)
Salaried/paid employment	45,247 (32.75)	34,608 (32.53)	61 (27.35)
Unknown	5473 (3.96)	3924 (3.69)	5 (2.24)
Education			
Primary or below	9407 (6.81)	5880 (5.53)	22 (9.87)
Secondary or equivalent	95,675 (69.24)	72,270 (67.93)	165 (73.99)
Tertiary or equivalent	29,931 (21.66)	26,106 (25.54)	34 (15.25)
Unknown	3160 (2.29)	2140 (2.01)	2 (0.90)
Area/community			
Rural	89,737 (64.95)	69,725 (65.53)	141 (63.23)
Urban	48,434 (35.05)	36,669 (34.46)	82 (36.77)
Unknown	2 (0.00)	2 (0.00)	0 (0)
Region			
Southern/South east district	60,102 (43.50)	47,458 (44.61)	92 (41.26)
North‐west district	16,088 (11.64)	12,444 (11.70)	20 (8.97)
Ghanzi (western) district	4652 (3.37)	3923 (3.69)	3 (1.35)
Central/North‐east district	57,329 (41.49)	42,569 (40.01)	108 (48.43)
Nationality			
Motswana	132,379 (95.81)	101,990 (95.86)	215 (96.41)
Non‐Motswana	5321 (3.85)	4104 (3.86)	8 (3.59)
Unknown	473 (0.34)	302 (0.28)	0 (0)
Gravida			
Primigravida	47,792 (34.59)	43,261 (40.66)	96 (43.05)
Multigravida	89,899 (65.06)	62,852 (59.07)	127 (56.95)
Unknown	482 (0.35)	283 (0.27)	0 (0)
Parity			
Nulliparous	51,757 (37.46)	46,580 (43.78)	102 (45.74)
Multiparous	83,262 (60.26)	58,091 (54.60)	119 (53.36)
Grand‐multiparous (>5)	2643 (1.91)	1422 (1.34)	2 (0.90)
Unknown	511 (0.37)	303 (0.28)	0 (0)

Note: Data are number (%) or median (IQR).

The number of repeat HIV tests was determined among women with a negative/indeterminate HIV test at initial ANC visit (*n* = 106,619); of these, 19,218 (18%) had one HIV test during pregnancy (including at delivery but before discharge), 53,496 (50%) had two tests and 33,628 (32%) had three or more tests. Less than 1% (*n* = 277) had an unknown number of tests. The proportion of participants with only one HIV test during pregnancy/delivery also differed by site (range 6–40%). Of women with an initial negative/indeterminate HIV test (*n* = 106,619), 103,529 (97%) also had a known conception date and at least one known test date allowing for the timing of HIV test(s) to be determined. Among these women, 78,162 (75%) had at least one test in the third trimester, including 9628 (9%) tested in the first, second and third trimesters; 43,319 (42%) tested in the second and third trimesters; 14,299 (14%) tested in the first and third trimesters; 10,916 (11%) tested in only the third trimester; and 1288 (1%) had a documented test date but this was at delivery. The median gestational age at first HIV test was 16 weeks (IQR 12, 22) and among women with at least two HIV tests, the median gestational age at last HIV test was 36 weeks (IQR 30, 38). Among women who had a first negative/indeterminate test in pregnancy and a known second test date, the median number of days between first and second tests was 102 (IQR 92, 123) days.

There were 223 seroconversions identified among 86,282 pregnancies (2.58/1000 pregnancies, 0.26%), with at least two known HIV test dates during pregnancy, yielding an IR of 0.69 (95% CI 0.60–0.78) per 100 person‐years. The majority of these women (*n* = 217) seroconverted after an initial negative test and six (3%) seroconverted after an initial indeterminate test. The median gestational age at date of diagnosis was 32 weeks (IQR 27, 36), calculated among 217 women with a known gestational age at delivery. The median time between the last negative/indeterminate HIV test and seroconversion was 98 (89, 128) days. For 177 (79%) of the 223 seroconverters, there was documentation of ART initiated during pregnancy and 68 (30%) had a documented start date of the initial regimen. Of these 68 women, 31 (46%) initiated ART on the date of their diagnosis and 55 (81%) within 7 days of their diagnosis. A total of 25,289 women did not have a documented HIV test in the third trimester. We estimate that approximately 58 (95% CI 51–66) seroconversions may have been missed during pregnancy due to a lack of repeat testing (i.e. 21% of total seroconversions, see Appendix 1 for calculations). In a secondary analysis, including second tests at or within 7 days after delivery as non‐missed, an estimated 33 (95% CI 28–37) seroconversions were missed among 15,695 women without an HIV test in the third trimester or within 7 days of delivery, or an estimated 13% of all seroconversions.

Prior studies from outside Botswana provide a range of 13–18% for *in‐utero* vertical HIV transmission risk among women who are recently infected [14, 15]. Using an estimate of 16% *in‐utero* transmission for unidentified seroconverters (*n* = 58), and a conservative estimate that this would be halved to 8% for identified seroconverters where ART was started by the early third trimester (*n* = 223), this would yield a total of 27 *in‐utero* transmissions among the identified and unidentified seroconverters in our study. In comparison, because of the high ART coverage, data from Botswana suggest only 0.4% *in‐utero* vertical transmission risk among women known to be living with HIV (either known from conception, or identified at the first test in pregnancy) [16]; applying this rate to the study population (*n* = 30,830) would yield 123 infections. Thus, incident infection may conservatively account for ∼ 18% (27/150) of the *in‐utero* vertical transmission risk in Botswana (see Appendix 1 for calculations).

In univariable logistic regression analyses, factors associated with seroconversion during pregnancy included younger age, lower educational attainment and not being married (Table 2). Women aged 35 years and older had lower odds of seroconverting in pregnancy compared to women aged 25–35 (unadjusted odds ratio [uOR] = 0.50; 95% CI 0.26–0.97). Women who were married had lower odds of seroconverting (uOR = 0.27; 95% CI 0.13–0.58) compared to women who were single. A primary or lower education level (uOR = 2.87; 95% CI 1.68–4.92) and secondary or equivalent education level (uOR = 1.75; 95% CI 1.21–2.54) were also associated with higher odds of seroconverting compared to a tertiary education level.

**Table 2:** Univariable analysis to identify factors associated with incident HIV during pregnancy among women in the Tsepamo study

	HIV negative at delivery	Women who seroconverted during pregnancy	uOR (95% CI)	*p*‐value
Age	*n* = 103,825	*n* = 223		
Under 15	208 (0.20)	1 (0.45)	2.52 (0.35–18.14)	0.360
15–24	49,177 (46.25)	123 (55.16)	1.31 (0.92–1.72)	0.159
25–35	46,568 (43.79)	89 (39.91)	Ref	Ref
35 and older	10,384 (9.77)	10 (4.48)	0.50 (0.26–0.97)	0.040
Marital status	*n* = 103,825	*n* = 218		
Single	91,366 (88.86)	209 (95.87)	Ref	Ref
Married	11,230 (10.92)	7 (3.21)	0.27 (0.13–0.58)	0.001
Widowed/divorced	229 (0.22)	2 (0.92)	3.82 (0.94–15.5)	0.060
Occupation	*n* = 102,472	*n* = 218		
Student	6858 (6.69)	12 (5.50)	0.74 (0.41–1.33)	0.308
Unemployed/housewife	61,006 (59.53)	145 (66.51)	Ref	Ref
Salaried/paid employment	34,608 (33.77)	61 (27.98)	0.74 (0.55–1.00)	0.050
Education	*n* = 104,256	*n* = 221		
Primary or below	5880 (5.64)	22 (9.87)	2.87 (1.68–4.92)	<0.001
Secondary or equivalent	72,270 (69.32)	165 (73.99)	1.75 (1.21–2.54)	0.003
Tertiary or equivalent	26,106 (25.04)	34 (15.25)	Ref	Ref
Area/community	*n* = 106,394	*n* = 223		
Rural	69,725 (65.53)	141 (63.23)	Ref	Ref
Urban	36,669 (34.47)	82 (36.77)	1.11 (0.84–1.45)	0.469
Region	*n* = 106,394	*n* = 223		
Southern/South east district	47,458 (44.61)	92 (41.26)	Ref	Ref
North‐west district	12,444 (11.70)	20 (8.97)	0.83 (0.51–1.35)	0.448
Ghanzi (western) district	3923 (3.69)	3 (1.35)	0.39 (0.12–1.25)	0.113
Central/North‐east district	42,569 (40.01)	108 (48.43)	1.31 (0.99–1.73)	0.058
Nationality	*n* = 106,094	*n* = 223		
Motswana	101,990 (96.13)	215 (96.41)	Ref	Ref
Non‐Motswana	4104 (3.87)	8 (3.59)	0.92 (0.46–1.87)	0.828
Gravida	*n* = 106,113	*n* = 223		
Primigravida	43,261 (40.77)	96 (43.05)	1.10 (0.84–1.43)	0.489
Multigravida	62,852 (59.23)	127 (56.95)	Ref	Ref
Parity	*n* = 106,093	*n* = 223		
Nulliparous	46,580 (43.90)	102 (45.74)	Ref	Ref
Multiparous	58,091 (54.75)	119 (53.36)	0.94 (0.72–1.22)	0.622
Grand‐multiparous (>5)	1422 (1.34)	2 (0.90)	0.64 (0.16–2.61)	0.536

Abbreviation: uOR, unadjusted odds ratio

## 4 DISCUSSION

Using data from the Tsepamo surveillance study, we found high rates of repeat testing in pregnancy and HIV incidence was low. However, an estimated 21% of total seroconversions may have been missed during pregnancy due to a lack of repeat testing in the third trimester (reduced to 13% missed seroconversions in total, when delivery testing at or within 7 days after birth was included).

Our estimate that incident infection may conservatively account for ∼ 18% (27/150) of the *in‐utero* vertical transmission risk in Botswana is higher than reported in the UNAIDS global stacked bar analysis which estimated that of 81,600 new child HIV acquisitions in 2018 during pregnancy, 13% were due to mothers acquiring HIV during pregnancy [17]. In settings, such as Botswana, where there is high HIV incidence but also high ART coverage for those known to be living with HIV, identifying acute HIV acquisition in pregnancy is critical to minimize vertical transmission.

The estimated HIV IR during pregnancy in this study (0.69/100 person‐years) was similar to the IR reported among participants (*n* = 18,597) in the Botswana Combination Prevention Project (BCPP) (0.71/100 person‐years) but lower than the reported IR among females only in the BCPP study (1.01/100‐person years) [18]. The BCPP study was a community‐randomized controlled trial conducted between 2013 and 2017 that measured HIV incidence in 30 rural or peri‐urban communities throughout Botswana, approximating HIV incidence in the general population (no specific IR in pregnant women was reported) [19]. While several prior studies have found that pregnant women have an increased risk of HIV acquisition [20, 21, 22, 23, 24], our data suggest that the incidence of HIV among pregnant women may be similar to the general population. There are several reasons why our IR might be lower than that found among females in BCPP. First, if we included our estimated missed seroconversions due to lack of repeat testing in the third trimester or within 7 days of delivery (*n* = 33), our IR increases to 0.79/100 person‐years. Second, we are underestimating the number of seroconversions during pregnancy as we could not include women who seroconverted during pregnancy but prior to presenting for ANC (in this case, their first HIV test in pregnancy would be positive). Third, our study was performed after the BCPP study, after Botswana had implemented the “test and treat” strategy and switched from efavirenz‐based to dolutegravir‐based ART. Therefore, it is possible that the overall incidence of HIV in Botswana was lower during our study period than during BCPP due to these interventions.

Our reported IR (0.69/100 person‐years) was also similar to that reported by Ortblad et al. (0.80 per 100 person‐years) in a recent study using programmatic data from HIV testing programmes at routine ANC clinics in Botswana. However, we found higher repeat HIV testing during ANC (>75% vs. 28%) and a lower proportion of documented ART initiation among women newly diagnosed with HIV (79% vs. 88%) [25]. Ortblad et al. report ART initiation among all women newly diagnosed with HIV (*n* = 853), while we investigated this among seroconverters only (*n* = 223). The differences in recorded ART initiation and repeat HIV tests reported between the two studies could also be due to our data being extracted from obstetric record cards, while Ortblad et al. extracted data from HIV testing registries at ANC clinics. Our finding that only 79% of women who seroconverted in our study had documented ART initiation during pregnancy is concerning as reported coverage of pregnant women who receive ART in Botswana is >98% [10]. While the estimated IR in our study was similar to that reported by other studies in Botswana [18, 19, 25], it was significantly lower than in a 2014 meta‐analysis [26], which reported a pooled HIV IR of 3.8 per 100 person‐years among pregnant/postpartum women in sub‐Saharan Africa; however, studies included in the meta‐analysis dated back to 1994, when HIV programmes were less established.

The BCPP study and Ortblad et al. identified female gender and younger age as risk factors for HIV seroconversion [18, 25]. In line with these findings, we found younger age to be associated with seroconversion during pregnancy with women aged 35 years and older having 50% lower odds of seroconverting compared to women aged 25–35 years. We also found lower levels of education associated with higher odds of seroconverting during pregnancy and being married associated with lower odds of seroconverting. Similar findings have been previously reported in other studies conducted in sub‐Saharan Africa among pregnant women and in the general population [27, 28, 29, 30]. Of note, occupation, region, nationality, gravida and parity were not associated with seroconverting in pregnancy.

Collectively, the findings of this study highlight the importance of reducing HIV incidence during pregnancy. Even with impressively high levels of repeat HIV testing during pregnancy at the national level (>70%), we still estimate that 21% of HIV seroconversions are missed in pregnancy, and only 8% of these are picked up at delivery (leaving 13% missed entirely). These missed events likely contribute to poor health outcomes for the mothers and undetected cases of paediatric HIV. Possible interventions to reduce the health impact of HIV seroconversion in pregnancy include the use of pre‐exposure prophylaxis (PrEP), regular testing of partners of pregnant women and increased frequency of testing during pregnancy. Currently, Botswana HIV guidelines recommend PrEP for individuals who engage in high‐risk sexual behaviour (e.g. sex workers), HIV‐discordant couples, individuals who cannot negotiate safe sex with their partners, people with multiple sexual partners and drug users [11]. However, in light of the findings in the current study, and given increased HIV susceptibility and risk of vertical transmission with incident HIV during pregnancy [26, 31], PrEP may be beneficial in HIV‐negative women of reproductive age. And while further research is required to establish safety and tolerability among pregnant women, a recent interim analysis among cis‐gender women found that long‐acting injectable cabotegravir was 89% more effective than oral PrEP (tenofovir/emtricitabine), offering a ground‐breaking tool to prevent HIV among women, particularly women with difficulties adhering to oral pills [32, 33].

The main strength of this study is the use of a large and complete database which represents a high proportion of all births in Botswana. Our study also has its limitations. First, our analysis was conducted using data from the Tsepamo birth outcomes surveillance study raising the possibility of bias associated with observational research using routine operational data, such as selection bias. Second, among women included in the timing of test analysis (*n* = 103,529), 5710 (6%) had at least one missing test date, therefore, it is possible that these women had an HIV test in another trimester of pregnancy that was not accounted for in the analysis. Our analysis also included third‐trimester tests all the way until the day before delivery, which possibly included testing while in labour; late third‐trimester re‐testing is unlikely to impact *in‐utero* transmission or to fully reduce the risk for intra‐partum transmission. Additionally, it is possible that a significant proportion of seroconversions during pregnancy were missed because we could not determine the timing of seroconversion for women who tested positive at their first HIV test during pregnancy (*n* = 5518); and HIV diagnosis in Botswana is made using third‐generation enzyme immunoassay screening tests, which cannot detect HIV acquisitions as early as fourth‐generation tests. Finally, our analysis was based on the assumption that IR is static over a pregnancy, which may not be the case.

## 5 CONCLUSIONS

Incident HIV occurred in 0.26% of susceptible women during pregnancy and likely accounts for a substantial proportion of vertical transmission in Botswana. Up to 21% of incident infections were undetected despite a high rate of re‐testing. To maximally decrease vertical transmission in countries with a high HIV prevalence and high ART coverage, such as Botswana, the detection and prevention of incident HIV acquisition during pregnancy should be prioritized.

## COMPETING INTERESTS

The authors declare no competing interests.

## AUTHORS’ CONTRIBUTIONS

RZ, RS, AM and GKM conceptualized the analysis. AM and RZ analysed the data and prepared the manuscript, and RS contributed to subsequent drafts. All authors critically reviewed and approved the manuscript.

## FUNDING

The Tsepamo study was funded by the US National Institutes of Health Funding (NIH/NICHD R01HD080471, R01HD095766), RZ was supported by K23 HD088230 and AM was supported by D43TW009610. The funding sources had no role in the design and conduct of the study; data collection, analysis, reporting; and decision to submit the manuscript for publication.

## Data Availability

Data available upon request.

## Appendix 1

### Calculations for missed seroconversions

**Primary analysis**—missed seroconversions among women without a test in the third trimester

Number of women who did not have a test in the third trimester = 25,289

Time between the last HIV test and delivery for 25,289 women = 8454.05 person‐years

Incidence rate = 0.69 per 100 person‐years

Missed seroconversions = $\frac{0.69\times 8454.05}{100}=$58

**Secondary analysis**—missed seroconversions among women without a test in the third trimester, at delivery or within 7 days of delivery

Number of women who did not have a test in the third trimester, at delivery or within 7 days of delivery = 15,695

Time between the last HIV test and delivery for 15,695 women = 4751.90 person‐years

Incidence rate = 0.69 per 100 person‐years

Missed seroconversions = $\frac{0.69\times 4751.90}{100}=$33

### Calculations for the estimated proportion of *in‐utero* vertical transmission risk due to incident HIV acquisition

Estimated *in‐utero* vertical HIV transmission risk among **women known to be living with HIV*** from a prior study conducted in Botswana = 0.4% [17]

*either known from conception or identified at the first test in pregnancy

Estimated *in‐utero* vertical transmission risk for identified seroconverters: = 8%

Estimated *in‐utero* vertical HIV transmission risk among **women with incident infection** from prior studies in Botswana = 13.4–17.8% [15,16].

*In‐utero* transmission risk for unidentified or “missing” seroconverters used in this analysis = 16% (average of 13.4% and 17.8%).

Number of seroconverters identified in our study = 223

Estimated number of *in*‐*utero* transmissions among identified seroconverters in our study:

8% × 223 = 17.84

Estimated number of *in*‐*utero* transmissions among the unidentified or “missing” seroconverters in our study:

16% × 58 = 9.28

Estimated total number of *in‐utero* transmissions in our study due to HIV acquisition during pregnancy:

17.84 + 9.28  = 27.12

Number of women in our study HIV positive at delivery but did not seroconvert during pregnancy = 30,830

Estimated number of *in‐utero* transmissions among non‐seroconverters in our study:

30,830 × 0.4% = 123

Estimated proportion of *in‐utero* vertical transmission due to incident HIV acquisition in our study:

$\frac{27}{123+27\;}=\;$18%
